# Biomechanical Aspects in Bone Tumor Engineering

**DOI:** 10.1089/ten.teb.2023.0106

**Published:** 2024-04-02

**Authors:** Ksenia Menshikh, Ivana Banicevic, Bojana Obradovic, Lia Rimondini

**Affiliations:** ^1^ Center for Translational Research on Autoimmune and Allergic Diseases, Università del Piemonte Orientale, Novara, Italy.; ^2^ Faculty of Technology and Metallurgy, University of Belgrade, Belgrade, Serbia.

**Keywords:** bone cancer, osteosarcoma, 3D in vitro models, bone tumor microenvironment

## Abstract

In the past decades, anticancer drug development brought the field of tumor engineering to a new level by the need of robust test systems. Simulating tumor microenvironment *in vitro* remains a challenge, and osteosarcoma—the most common primary bone cancer—is no exception. The growing evidence points to the inevitable connection between biomechanical stimuli and tumor chemosensitivity and aggressiveness, thus making this component of the microenvironment a mandatory requirement to the developed models. In this review, we addressed the question: is the “*in vivo – in vitro*” gap in osteosarcoma engineering bridged from the perspective of biomechanical stimuli? The most notable biomechanical cues in the tumor cell microenvironment are observed and compared in the contexts of *in vivo* conditions and engineered three-dimensional *in vitro* models.

**Impact statement:**

The importance of biomechanical stimuli in three-dimensional *in vitro* models for drug testing is becoming more pronounced nowadays. This review might assist in understanding the key players of the biophysical environment of primary bone cancer and the current state of bone tumor engineering from this perspective.

## Introduction

Osteosarcoma is a type of primary bone cancer found among children and older people, having a bimodal age distribution in the human population.^1^ It is estimated that 3–4 million people are diagnosed with this disease every year, which places osteosarcoma in the category of rare tumors. Still, osteosarcoma is recognized as a huge burden to society as it is an aggressive tumor with a high tendency for metastasis both in the earlier and later stages of the disease, most often in the lungs.^1,2^ In addition, osteosarcoma is an insidious tumor that is not preceded by benign precursor lesions, revealed due to local pain, and is diagnosed as high grade in most cases.^1,3^

Modern strategy for osteosarcoma treatment includes surgical resection of the primary tumor, complemented by chemotherapy. Given that sarcomas are of mesenchymal origin, and the tumor does not form a confined area as carcinomas, resecting the tumor with clear margins may be challenging and lead to local recurrence.^4^ Adapting the existing technologies—such as application of Raman spectroscopy for live imaging of single osteosarcoma cells—may increase the chance of precise excision.^5^ With surgical intervention only, the 5-year survival rate in patients is only 10–20%, whereas with incorporated chemotherapy it raises to 50–70% in case the tumor is localised.^6^ In patients who develop metastatic disease, the survival rate is only 20–30%.^7^

Circumstances that render osteosarcoma investigation difficult are—along with the low incidence rate in the human population with sudden occurrence and absence of precursor lesions—also the genomic instability and histological heterogeneity.^8^ To overcome these challenges there is a need for more advanced approaches and physiologically relevant models, which would significantly improve both osteosarcoma research and development of efficient therapies.

The development of innovative antitumor treatments requires reliable testing methods, as well as advanced models, at the stage of preclinical trials. Nowadays, models for screening the drug effectiveness *in vitro* include human tumor cell cultures in two-dimensional (2D) and three-dimensional (3D) cell culture systems, where the latter are becoming more widely used thanks to their similarity to physiological conditions of tumor development.^9^ However, most studies focus mainly on modeling soft tissue cancers, such as prostate and breast cancers. Bone, unlike soft tissues, possesses remarkable stiffness and high mineral content. Therefore, there is a need of setting up a robust, tuneable, and reproducible *in vitro* model of bone cancer.^10^

Acknowledging the role of the tumor cell microenvironment in encouraging and guiding cells toward pathological behavior, the scope of this review is to investigate to what extent biophysical stimuli are incorporated into different osteosarcoma 3D *in vitro* models.

## Osteosarcoma Microenvironment

Osteosarcoma cells are thought to arise from mesenchymal stem cells or progenitor cells during the obstructed differentiation into osteoblasts.^11^ Around 80% of all osteosarcoma cases, as well as an overwhelming majority of bone metastases, originate in the red bone marrow which finds a niche mostly in cavities provided by a spongy architecture of trabecular bone.^12,13^ Thus, we will be mainly focusing on the properties of this type of bone tissue, underlining that, in general, bone marrow is a heterogenic structure and demonstrates different parameters throughout its volume within one bone.^14^

The key features of the bone microenvironment playing role in the fate of the tumor include complex interactions between cells inhabiting the bone, a highly organized vascular network with the potential for neovascularization, hypoxic conditions, acidic environment, contributing to osteolysis processes, specific matrix composition with around 30% of mineral component and 70% of collagen, high calcium level, and remarkable mechanical properties.^13^ In addition to the above list, it should be noted that bone is a mechanically active microenvironment since it is commonly subjected to mechanical loadings during everyday activities. Mechanical loadings provide biomechanical stimuli to the bone resident cells and have a significant role in bone physiology, remodeling, repair, and tissue homeostasis.^15^ Among biomechanical stimuli, the most prominent are compression, compression-induced strain (substrate deformation), and fluid flow-induced shear stress.^16^

Solid tumors are tissues densely packed with both tumor and stromal cells within the tumor extracellular matrix. The interstitial structure in a tumor is less organized than in the surrounding healthy tissue, and the fibroblasts in the tumor stroma are present in high numbers.^17^ In particular, osteosarcoma histology shows pleomorphic tumor cells surrounded by osteoid which is a nonmineralized collagen-rich tissue characteristic for immature bone.^18^ Osteoid production is one of the characteristic features of osteosarcoma cells being also used in detecting the disease.^19,20^

With time, tumors increase in volume because of the characteristically fast proliferation of tumor cells.^17^ Tumors—and osteosarcoma is no exception—are permeated by a continuously developing network of vascular vessels with high permeability and irregular branching serving to supply tumor cells with adequate quantities of nutrients. In addition, the vascular network plays a key role in tumor cell intravasation and extravasation which is related to tumor metastasis and drug resistance.^21^

On a cell-scale level, multiple components are shaping its environment. Studies from past decades pointed to the importance of the tumor cell microenvironment in tumor progression, metastases, and invasion thus switching the tumor cell-centered view to the cell microenvironment, acknowledging its contribution also to the drug resistance.^22^ *In vivo* tumor cell microenvironment comprises cells of different types, extracellular matrix, signaling molecules, and a variety of biomechanical forces acting on the cells. It is a dynamic system in which distinguishing each factor and its contribution triggering cell response and influencing cell behavior is particularly challenging due to their mutual interactions with synergistic or even nullifying effects.

**FIG. 1.**
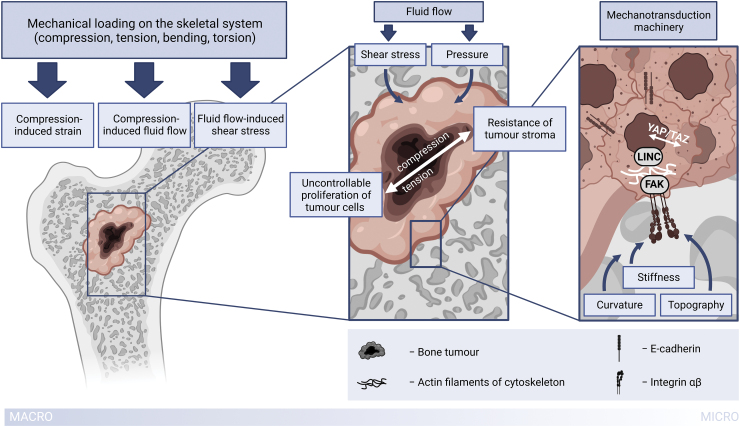
Principal scheme of biophysical components of the osteosarcoma microenvironment. Created with the use of BioRender.com Color images are available online.

Summarizing all of the above, components present in bone tumor cell microenvironment can be divided into biochemical (surrounding cells; pH, oxygen, and nutrient gradients; various soluble factors) and biophysical (extracellular matrix properties and external biomechanical and biophysical stimuli).^21^ We will be reviewing the latter in the context of osteosarcoma. The principal scheme of biophysical components of the osteosarcoma microenvironment is presented in Figure 1.

## Methods of Measuring Tumor Mechanical Properties

Measuring the mechanical properties of bone tumors holds great potential to assist in our understanding of tumor biomechanics and facilitate its translation into creating relevant *in vitro* bone tumor models.

Many studies focus on mechanical properties of osteosarcoma cells alone and mechanotransduction pathways in the cells (e.g., as reviewed in Muller and Silvan, 2019).^23^ Atomic force microscopy and microindentation techniques are mostly applied, which could be further supplemented with traction force microscopy to assess cell deformability and traction force generation.^24^ To assess mechanical properties of free-floating cells relevant for metastatic dissemination of cancer cells real time deformability cytometry is used.^23^ However, studies on *in vivo* osteosarcoma tumor mechanical properties are scarce and here we will briefly discuss methods for assessing the values of mechanical cues *in vivo* that we have reviewed in this article. Laboratory-based measurements of local tumor stiffness commonly involve *ex vivo* indentation techniques such as already mentioned atomic force microscopy or microindentation.^25,26^

**FIG. 2.**
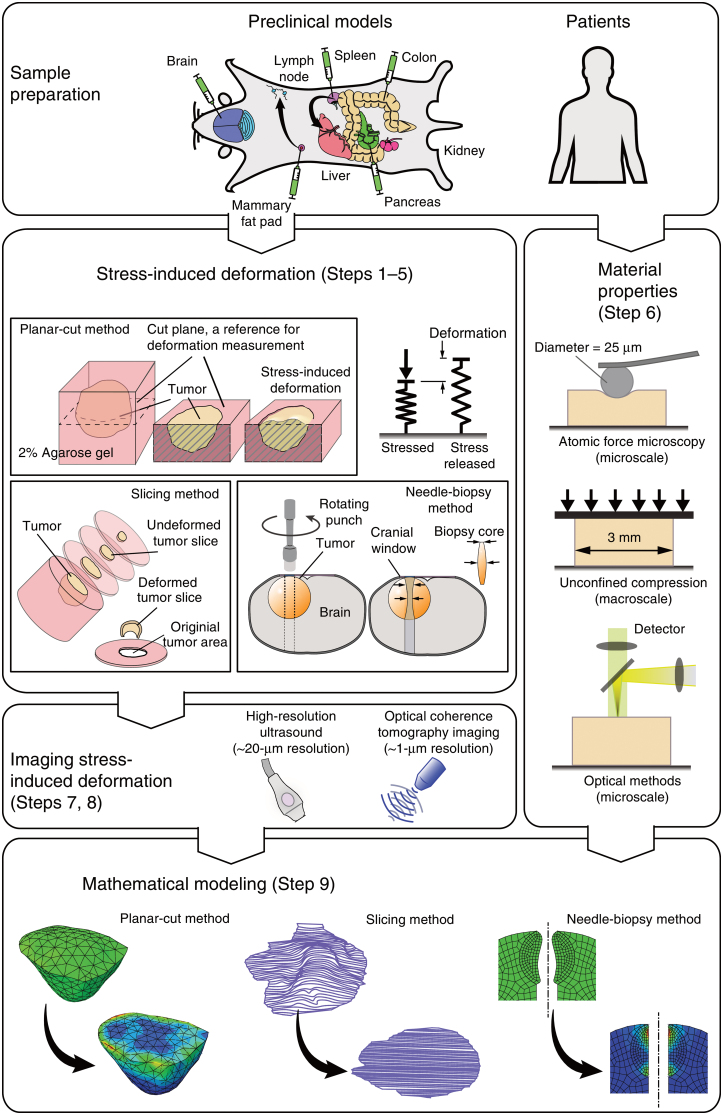
Overview of the methods used to evaluate solid stresses in tumors. Adopted from Nia et al.^29^ with permission from the publisher (License number: 5618840968051). Color images are available online.

Quantification of solid stress in tumors has been a subject of mathematical modeling, which has so far provided valuable resources for introducing this stimulus in bone tumor studies.^27,28^ Notably, experimental measuring of solid stress has been recently advanced by introducing novel techniques which include the planar-cut method for 2D spatial mapping of solid stress, the slicing method for sensitive measurements of stiffness in small tumor areas, and the needle-biopsy method for *in situ* solid stress quantification.^29^ Overview of the methods used to evaluate solid stresses in tumors is shown in Figure 2. These methods could offer new avenues for bone tumor investigations as well.

Determination of interstitial flow-induced shear stress *in vivo* remains a challenging task mostly due to the low velocity of interstitial fluid and the heterogeneous structure of the tumor.^30^ One of the approaches for interstitial fluid flow velocity determination *in vivo* was based on using fluorescence recovery after photobleaching in rabbits.^31^ Recently, significant advancement in this area has been accomplished by introducing a novel noninvasive magnetic resonance imaging (MRI) technique named convection-MRI. This technique enables measuring low-velocity fluid flow in solid tumors.^32^

## Biophysical Factors in Osteosarcoma Microenvironment

### Biomechanical stimuli: how do cells feel them?

Biomechanical stimuli, as a part of the cell biophysical environment, are exerted directly at the cell surface, and as a result, cell structure suffers from subtle deformations in the order of Angstroms.^33^ The cells sense and respond to biomechanical stimuli due to the prior conversion of these stimuli into biochemical signaling in the process known as mechanotransduction.

The first “layer” of the mechanotransduction apparatus is composed of integrins. In the case of bone tissue cells, integrins are not the only known mechanosensors that can be met: other structures, such as primary cilia or membrane caveolae, also contribute to the process of sensing with their specific molecular pathways.^34^ Integrins are connected to the matrix outside of the cell and to the complex of proteins inside, and, together, this combination orchestrates under the general term—focal adhesion complex. The configuration, quantity, and presence of participants in this “orchestra” are defined by the matrix itself and the cell affinity for the matrix. The focal adhesion complex is connected to actin filaments, which together with other components, form the cytoskeleton of the cell. The next “layer” is aimed to deliver the signal from the cytoskeleton to the nucleus interior and this is executed mainly by shuttling proteins YAP and TAZ, as well as LINC—linker of nucleoskeleton and cytoskeleton.^35^

Thus, cells are equipped with mechanoreceptor machinery so as to adequately gather information concerning the changes in their microenvironment.^33^ It is important to underline that mechanosensation and cell signaling can be modified or interrupted by defective mechanotransduction, which can be the starting point of pathological occurrences in bone.^36^

**FIG. 3.**
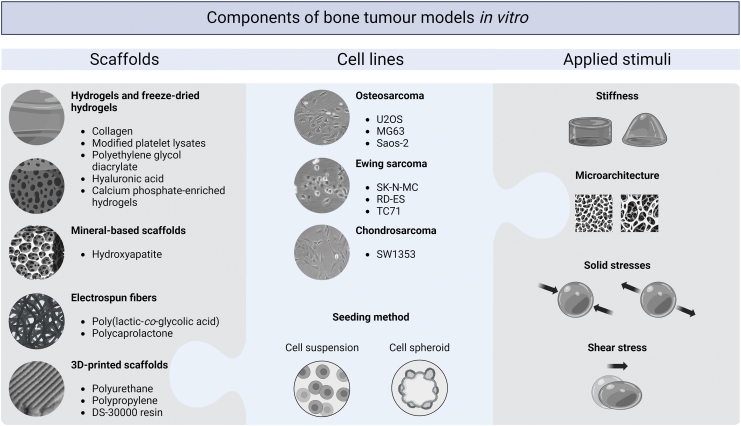
Examples of scaffolds, cell lines, and applied stimuli utilized in the reviewed *in vitro* models of primary bone cancers. Created with the use of BioRender.com Color images are available online.

Approaches to the development of bone tumor models *in vitro* utilizing biomechanical stimuli are reviewed in the following sections and are schematically summarized in Figure 3.

### Stiffness

High heterogeneity of trabecular bone architecture makes this type of tissue hard to analyze. For example, Young's modulus ranges between 10 and 3000 MPa and yield strain in compression ranges from 0.70% to 0.77% depending on the anatomic site (the measurements were performed on hydrated specimens considering anatomical and material axes).^37–39^ Although the same architecture that determines strain in the tension of trabecular tissue varies from 0.65% to 0.71%, elastic modulus is around 10–20 GPa.^37,39^ This can be partly explained by the difference between the two main phases of bone tissue—stromal and mineral.^19^ Needless to say, the mechanical properties stated above will differ on micro- and nanoscales.^34,40^

As for the pathological state, in an overwhelming majority of cancer cases, the tumor matrix tends to be of an increased stiffness compared to normal tissues thereby providing more opportunities to build focal adhesion points and, consequently, allowing the tumor to grow. On the opposite, osteosarcoma is known to soften the surrounding matrix from 2 to 14 GPa to >689 MPa.^41^ Thus, stiffness is considered one of the first features present in pathological physiological conditions to pay attention to when developing a microenvironment *in vitro.*^42^

Charoen et al. conducted research comparing the viability of spheroids grown from breast cancer cells and osteosarcoma cells depending on the stiffness of the gel in which they were embedded. The authors chose a wide variety of collagen gel concentrations and showed that osteosarcoma cells “prefer” a stiffer environment compared to softer, which in turn was preferred by breast cancer cells, and demonstrated high metabolic activity and remarkable growth in the gel of their choice.^43^ Monteiro et al. used methacryloyl platelet lysates (PLMA) scaffold for constructing an *in vitro* model consisting of osteosarcoma cells, as well as osteoblast and mesenchymal cells.^44^ Before that study, they defined the PLMA of an optimum stiffness with the Young's modulus of 15 kPa providing both the sufficient viability of mesenchymal stem cells and better invasiveness of tumor spheroids along with the formation of necrotic core and middle layer of quiescent cells.^45^

Another trial of recapitulating the tumor complexity was undertaken by Bassi and coworkers.^46,47^ They compared the cell-biomaterial interaction in two models—a hydroxyapatite-enriched collagen scaffold and a fully hydroxyapatite-based porous scaffold, both with osteosarcoma spheroids entrapped within. The spheroids without any scaffolds were also analyzed, and a 2D conventional cell culture was used as a control. The scaffolds were of quite different stiffness values: 30.93 ± 6.14 kPa for the collagen based with an opposite of 1.8 ± 0.2 GPa for the hydroxyapatite-based scaffold. The focus of the study was on the tumor stemness, which is strongly related with tumor malignancy. The expression levels of stemness markers (in this study—NANOG, OCT-4, HIF-1α, NOTCH-1, IL-6) were significantly higher in spheroids within scaffolds than without any. But low-stiffness and high-stiffness scaffolds influenced differently the two osteosarcoma cell lines used in this research showing higher stemness of Saos-2 cells in stiffer ones and of MG63 cells in softer ones.^46^

In another study on tumor stemness, polyethylene glycol diacrylate hydrogels were seeded with tumor cells of different types to show the “preferences” of the cells to various stiffnesses depending on the tissue of origin. Markers of colonization potential were used to analyze this inclination. As expected, osteosarcoma U2OS cells had these markers upregulated on a stiffer gel of 50 kPa, while, for example, breast cancer cells MCF7 on a 5 kPa gel.^47^

### Microarchitecture

In bone tumor engineering, not only mechanical characteristics of the material play an important role but also its microstructure. Modern technologies, such as additive manufacturing, allow approximating the unique architecture of native tissues. For instance, one study used micro computed tomography (μCT) images of femoral epiphysis to design *in silico* model for printing a bone-like scaffold. Printed by stereolithography, transparent biocompatible scaffolds simulated trabecular bone architecture precisely and were successfully colonized by mesenchymal stem cells. Cell-seeded scaffolds were then used as a bone-like niche for mimicking breast cancer metastasis. Chemosensitivity of cancer cells residing in such matrix was significantly lower than of those cultured on commercially available Matrigel, while drug response closely repeated the average clinical outcomes underlining the importance of the “right” microenvironment.^48^

In another study, the flexibility of 3D printing was utilized to achieve the optimal mechanical parameters and architecture. By varying the distance and the angle between the filaments of printed polyurethane lattice, it was possible to obtain the scaffolds not only with Young's modulus in the desired order of magnitude of MPa but also with the porosity enabling efficient cell colonization. Subsequently, mesenchymal stem cells were cultured on the obtained scaffolds for 3 weeks and then removed with the maintenance of the key extracellular matrix components. Such “biohybrid” scaffolds were successfully used as a microenvironment for Saos-2 cells.^49^

At this moment, general rules on the curvature thresholds that can be sensed by cells are not defined yet. *In vitro* studies show that there exist types of cells sensing curvature within the range of their size, as well as cells able to feel curved surfaces with a diameter exceeding 1 mm and larger. Along with stiffness and elasticity, convex and concave surfaces can alter YAP/TAZ signaling pathway affecting the cell shape.^50^ In bone, such curved microstructures are represented by osteons. The osteons resemble cylinders of irregular forms with a diameter in the range of 233 ± 23 μm in young humans and are organized on the nanoscopic level mostly by collagen type I and apatite minerals permeating the organic matrix.^51^

Electrospinning methods are used frequently to mimic the bone microenvironment from this perspective. In one study, a fibrous mesh made up of poly(lactic-co-glycolic acid) electrospun fibers of 10 and 30 μm in diameter was used for coculturing of osteosarcoma and endothelial cells showing cell attachment, cell–cell interactions, and development of lumens by endothelial cells.^52^ The attachment of cells and formation of vessel-like structures indicate a well-designed scaffold in terms of its microstructure.

### Solid stresses

Solid stresses are mechanical forces exerted on a unit area by nonfluid tumor components^53^ and encompass compressive and tensile stresses generated by the uncontrollable proliferation of tumor cells in conjunction with the resistance of stroma. It is estimated that compressive stresses in human tumors fall in the range from 4.7 to 18.9 kPa.^53^ As a result, tumor cells are compressed, which was shown to affect their behavior.^54^ Even low-magnitude compression *in vitro* (50 to 295 Pa for different periods up to 24 h) led Saos-2 osteosarcoma cells to behave differently compared to the nonstimulated group and to produce a higher amount of matrix proteinases.^54^

Another direct consequence of solid stress is extracellular matrix straining, which consequently causes radial and circumferential tensile stresses on the surrounding tissue.^55^ Tensile forces were also shown to have an impact on osteosarcoma cell behavior. Application of mechanical strains on osteosarcoma cells (3 cycles/min, 5000 μstrain on average, 1–4 days) demonstrated that 4 days of exposure to mechanical stimuli alone, without hormonal influence, can promote the expression of collagen I, osteocalcin, and osteopontin.^56^ Other studies found integrin-β overexpression in mechanically stimulated cells performed by subtle cyclic deformations of a substrate.^57,58^ Interestingly, osteosarcoma cells are not only sensitive to the intensity of tensile forces but also to the direction of their action,^59^ and even the relationship between tensile forces and osteosarcoma metastasis was observed.^60^

Incorporating compression and tensile forces in 3D *in vitro* models is most commonly achieved using bioreactors with dynamic compression that imitate the existence of these stimuli *in vivo*. Due to the scarcity of studies concerning compression and strain effects on 3D cultured osteosarcoma cells, in the following text, we will present studies focusing on Ewing sarcoma cells instead, which can still shed more light on our understanding of the whole process chain thanks to similarities of these types of primary bone cancer.

Mechanical loadings are found not only to regulate tumor cell functions as they do in normal cells but also to promote tumor cell invasiveness.^61^ Marturano-Kruik et al. as well confirmed this finding by involving mechanical loading in their 3D *in vitro* model of Ewing sarcoma. The model was based on bioengineered bone, obtained by human mesenchymal stem cell cultivation on decellularized bone scaffolds, and infused with Ewing sarcoma spheroids upon development.^62^ The cultures were subjected to three cycles of loading (0.7% strain, 1 Hz, 30 min of stimulation) per day. Depending on the piston position, stress magnitudes varied within the construct and were in the range from 2.5 × 10^5^ to 5 × 10^5^ Pa. The study showed that introducing biomechanical stimuli in the form of cyclic strains activated mechanotransduction machinery in Ewing sarcoma cells leading to the development of a more aggressive tumor phenotype.^62^

The same research group extended their study to investigate the effect of mechanical stimulation on the chemosensitivity of Ewing sarcoma cells. Cells were seeded into scaffolds prepared from collagen I and hyaluronic acid and were subjected to mechanical stimulation (1% strain, 0.25 Hz, 2 h of stimulation per day). Even though perfusion through the construct was absent in this model, the cells were exposed to fluid shear stresses, which were indirectly derived from mechanical loading. Mechanical loading coupled with shear stresses activated signaling mechanotransduction pathways, which promoted Ewing sarcoma cells to exhibit increased drug resistance.^63^

### Shear stress

Shear stresses in tumors derive from fluid flow (interstitial fluid, blood, or lymph) and act upon the cell surface. Shear stress magnitudes generally depend on fluid velocity and fluid viscosity and therefore vary across the tumor interior—fluid flow itself is not steady meaning that the fluid velocity is changing with time, and in addition, fluid velocity depends on the size of pores between the cells and extracellular matrix. In solid tumors, however, shear stress is thought to be low because the cells are well protected by matrix.^53^ Although difficult to determine exact shear stress magnitudes, it was estimated that the average interstitial fluid flow-induced shear stress on tumor cells is 0.01 Pa.^64^ We assume that this value can be translated to osteosarcoma.

The physiological range of shear stresses can regulate cell cycle and differentiation. By applying 1.2 Pa shear stress on osteosarcoma (MG63, Saos-2) and chondrosarcoma (SW1353) cell monolayers, it was found that it induced G2/M cell cycle arrest.^30^ A more recent study of the same research group found that high shear stresses of 2 Pa induced upregulation of stearoyl-CoA desaturases-1 (SCD-1) levels in human osteosarcoma MG63 cells, which may have an auto-protective role in cell survival. In fact, osteosarcoma cells with genes silenced for SCD-1 expression experienced death in larger number when subjected to high shear stresses.^64^ Flow-induced shear stress of 1 Pa upregulated YAP protein in cells silenced for expression of Sox, a gene that regulates YAP expression. YAP is a protein with a key role in bone development and homeostasis, responsible to some extent for musculoskeletal tumorigenicity. The same study confirmed that shear stress can alter osteogenic gene expressions and ultimately guide cells toward their normal program of differentiation.^65^

From the mentioned studies, it can be observed that shear stresses lower than 1.2 Pa induce osteosarcoma cell death, while surprisingly higher magnitudes have the opposite effect. However, these investigations were performed on cell monolayers, and a definite conclusion should be drawn after introducing shear stresses to physiologically more relevant osteosarcoma models.

Shear stress was introduced as a biomechanical signal in 3D *in vitro* models for osteosarcoma cell cultivation in several studies. In one study, murine K8 osteosarcoma cells attached to a collagen foam were cultivated under perfusion conditions (medium flow rate 1.3 mL/min, corresponding to the superficial velocity of 276 μm/s average shear stress 1.57 × 10^−4^ Pa) for 21 days. Medium perfusion had beneficial impacts on osteosarcoma cells, which proliferated more and expressed more collagen type I and osteocalcin. Still, the perfusion did not have an impact on osteopontin expression. In this study, shear stress was thought to have a negligible effect on osteosarcoma cell morphology and activity. However, a combination of shear stress with enhanced mass transport succeeded to some extent in mimicking the bone marrow cavity environment, the place where osteosarcoma initially occurs.^66^

A similar effect of medium perfusion and shear stress acting on osteosarcoma cells was observed in a different study.^67^ Osteosarcoma Saos-2 cells were seeded on polyurethane porous scaffolds and cultured in a perfusion bioreactor (medium flow rate 3 mL/min, corresponding to the superficial velocity of 4000 μm/s, calculated shear stress 0.06 Pa). The study reported similar results as the abovementioned study with the difference in increased osteopontin levels in perfused cultures.^67^ It is difficult to assume the possible cause of the osteopontin increase since it could have been caused either by enhanced mass transport or higher shear stress.

To uncouple the contribution of shear stress and mass transport on cell behavior, shear stress can be independently varied while the mass transport rate is held constant, which can be achieved by utilizing media of different viscosities while maintaining the same fluid flow rate.^68^

This approach of separating shear stress and mass transport influences was applied to investigate the effect of shear stress on Ewing sarcoma cell sensitivity to Insulin growth factor-receptor (IGF-1R) inhibitors. These inhibitors can be potentially used in Ewing sarcoma treatment, given the fact that the IGF1/IGF-1R signaling pathway plays a significant role in Ewing sarcoma malignancy. A 3D model of Ewing sarcoma was obtained by seeding cancer cells on poly(ɛ-caprolactone) scaffolds and cell cultivation under medium perfusion. First, it was observed that the perfusion upregulated IGF1 production proportionally to the flow rate. Moreover, with increased perfusion, cells appeared to be more sensitive to IGF-1R inhibitors owing to enhanced mass transport. However, the same perfusion flow rate (0.2 mL/min) and different values of shear stress (1.7 × 10^−3^–17 × 10^−3^ Pa) revealed shear stress dependency of both IGF1 production and cell sensitivity to IGF-1R inhibitors: higher shear stresses led to the increased production of IGF1 and lower sensitivity of Ewing sarcoma cells to IGF-1R inhibitor.

This observation is another strong implication that drug efficiency evaluation should be conducted using an adequate 3D model with incorporated biomechanical cues so as to avoid discrepancies between clinical and *in vitro* results.^69^ Likewise, another study confirmed that IGF1 production by Ewing sarcoma cells is shear stress dependent but a negligible effect of flow on IGF-1R secretion was observed.^70^ In that study, to imitate the heterogeneous tumor structure, polypropylene fumarate scaffolds were 3D printed with pore size gradients. The scaffolds were seeded with cells and perfused at a 0.6 mL/min flow rate (corresponding to the superficial velocity of 127 μm/s). Distribution of shear stresses throughout the scaffold was determined by computational modeling, and the magnitudes were up to 8 × 10^−3^ Pa.

## Osteosarcoma Treatment Strategies Inspired by Cancer Biomechanics

Current therapeutic strategies targeting OS biomechanics can be divided into those directly affecting molecular cues of mechanotransduction and those acting indirectly by influencing bone remodeling processes or alternating biophysical stresses. In Table 1 we present several biomechanics-inspired treatment strategies reviewed in the recent literature analyses.^71–73^

**Table 1.** Treatment Strategies Targeting Osteosarcoma Biomechanics

Strategy	Target	Compound	Evidence	Reference
Targeting mechanotransduction pathways	Ezrin - connects the actin cytoskeleton and plasma membrane	NSC305787 and NSC668394	Inhibit lung metastasis of ezrin-sensitive OS cells injected in a mouse model	^82^
		NSC305787	Inhibits OS lung metastasis in a transgenic mouse model	^83^
	Integrin αvβ3 - transmits signals from the physical environment; of the integrin family, it plays the biggest role in angiogenesis	Combination of Cyclo (RGDyK) and Timosaponin AIII	Inhibits the metastatic activity of OS cells *in vitro* in transwell insert model and *in vivo* in OS cell-injected mice	^84^
	YAP/TAZ and related molecules	Verteporfin	Downregulates the expression and activity of YAP, slows down OS cell proliferation and migration *in vitro*	^85^
		Pazopanib	Inhibits tyrosine kinase VEGFR activity; 60% and 68% of patients in 2 independent studies show clinical benefit	^86,87^
		Dasatinib	Inhibits Src tyrosine kinase activity; Impacts survival and migration of Src-dependent OS cell lines *in vitro*	^88^
		Simvastatin	Inhibits OS cell proliferation and induces apoptosis *in vitro*	^89^
			Induces OS cell apoptosis *in vitro*; in combination with metformin inhibits tumor growth *in vivo*	^90^
	IGF/IGF-1R signaling pathway	Dominant-negative IGF1R (dnIGF1R) mutants	Blockade of IGF/IGF-1R signaling axis; Inhibition of cell migration and progression of cell cycles in OS cells	^91^
	IGF-1R/mTOR signaling pathway	Cixutumumab/temsirolimus combination	Antitumor activity; Inhibition of IGF-1R and mTOR pathways; In clinical studies	^92^
Targeting bone structure and remodeling	Mineralized bone matrix	Pamidronic acid-functionalized nanoparticles with doxorubicin	Antitumorigenic activity in combination with doxorubicin in mice and dogs	^93^
		Zoledronic acid	Antitumorigenic and antiangiogenic activity *in vitro* (OS and endothelial cells) and *in vivo* in a mouse model	^94^
			57.1% event-free survival (63.4% in the control group) in phase III clinical trial in combination with chemotherapy	^95^
	RANKL	mRANK-Fc	Prevented formation of osteolytic lesions, tumor growth, and metastasis in a mouse model	^96^
		OPG	Prevented formation of osteolytic lesions, reduced tumor incidence, and local tumor growth in mice and rats	^97^
		Rkl-siRNAs	Protected from osteolysis; in combination with ifosfamide blocked tumor progression in a mouse model	^98^

Based on the recent review articles by Shoaib et al., Belayneh et al., Luu and Viloria-Petit.^71–73^

IGF-1R, insulin growth factor-receptor; OS, osteosarcoma.

As can be seen, especially in the example with bisphosphonates (pamidronate and zoledronate) which are able to bind to the mineralized bone matrix, clear treatment strategy and promising results *in vitro* and *in vivo* do not guarantee the positive outcome in clinical trials.^73^ In the case of the agents targeting bone remodeling, it can be explained by the heterogeneity of the bone tumor microenvironment having both regions with abnormal resorption and regions with abnormal growth.^72^

For now, the ongoing research points to the necessity of combined therapies. For instance, not only in the context of osteosarcoma but also for the majority of cancers with solid malignancies it has been shown that alleviating solid stress in tumors can increase the drug efficacy by decompressing blood and lymphatic vessels and enabling a more efficient drug perfusion.^74^ Increased interstitial pressure in a tumor may also act as a target of manipulation,^75^ as well as unusual stiffness of the tumor can potentially be used as a biomechanical biomarker.^76,77^ Diverse strategies for reducing matrix stiffness have been extensively reviewed as a therapeutic approach for solid tumors.^78–80^ Nevertheless, this approach should be taken with caution given the unintended consequences such as facilitating tumor cell migration and fostering its invasiveness. Conversely, in the context of osteosarcoma cells, an *in vitro* study has shown that a soft matrix enhances stemness and drug resistance, whereas a stiffer matrix supports osteosarcoma spread, migration, and proliferation.^81^ This implies the complexity of using stiffness as a mechanical cue for a therapeutic target.

## Conclusion and Future Perspectives

In this review, we took an effort to look at the challenging task of osteosarcoma engineering from the perspective of biomechanics and mechanobiology. From reviewing the literature, the most prominent biomechanical cues in the tumor cell microenvironment emerged to be the extracellular matrix stiffness, compression stress, tensile stress, and fluid flow-induced shear stress. These stimuli simultaneously act on tumor cells, and their net effect proves to be crucial in an orchestrated chain of oncogeneses, progression, and metastasis contributors.

Three dimensional *in vitro* osteosarcoma models, a result of osteosarcoma engineering, are becoming increasingly acknowledged by the scientific community as being more relevant for disease study and antitumor drug testing in comparison to their counterparts—traditional cell monolayers and animal models. Still, nowadays the incorporation of biomechanical stimuli in osteosarcoma models is mainly lacking, but according to the studies that are reviewed in this article, biomechanical stimuli appear to be crucial in the activation of the same mechanotransduction pathways participating along the tumor progression timeline as *in vivo*. Therefore, the presence of biomechanical stimuli *in vitro* renders osteosarcoma models more complex and at the same time more relevant. We summarized the main highlights of the observed literature in Table 2.

**Table 2.** Elaboration of Biophysical Stimuli in Bone Cancer *In Vitro* Three Dimensional Models

Biophysical stimuli	Stimulus numerical value in vivo*^a^*	Stimulus numerical value in vitro	Method of introducing stimulus in vitro	Cell line*^b^*	The observed influence of a stimulus in a 3D in vitro model	References
Stiffness	> 689 MPa^41^	From 10 to 200 kPa	Collagen gels of concentrations from 2 to 5 mg/mL	Osteosarcoma U2OS and breast adenocarcinoma MDA-MB-231	Higher U2OS cells' metabolic activity on stiffer gels	^43^
		15 kPa	PLMA hydrogel	Osteosarcoma MG-63 alone or with bMSCs and fetal osteoblasts FhOBs	Invasiveness of MG-63 tumor spheroids, formation of the necrotic core when in coculture on a scaffold	^45^
		2 GPa	HAp or HAp-collagen scaffold	Osteosarcoma MG-63 and Saos-2	Higher stemness of SaOS-2 cells on a stiffer (HAp); higher stemness of both when cultivated on scaffolds than in monolayer	^46^
		50 kPa	PEGDA hydrogel	Osteosarcoma U2OS and breast cancer MCF7	Upregulated markers for colonization potential for U2OS cells on a stiffer (50 kPa) gel and for MCF7 cells on a softer one (5 kPa)	^47^
		>2 MPa	3D-printed polyurethane scaffolds, stiffness depends on lattice parameters	Osteosarcoma Saos-2 and MSCs	Better cell colonization and proliferation on the scaffold compared to softer ones (< 2 MPa)	^49^
Compression stress	4.7–18.9 kPa^54^	250–500 kPa (0.7% strain, 1 Hz, 3 × 30 min/24 h)	Bioreactor with dynamic compression	Ewing sarcoma SK-N-MC and MSCs	Development of more aggressive tumor phenotype	^62^
		1% strain, 0.25 Hz, 1800 cycles of total 2 h/24 h	Bioreactor with dynamic compression	SK-N-MC, 140 RD-ES, and patient-derived Ewing sarcoma cells	Increased drug resistance of tumor cells	^63^
Shear stress	10 mPa^103^	0.16 mPa	Perfusion bioreactor	Murine osteosarcoma K8	The influence of shear stress is not clear and distinguished from the others	^66^
		60 mPa	Perfusion bioreactor	Osteosarcoma Saos-2	The influence of shear stress is not clear and distinguished from the others	^67^
		1.7–17 mPa	Perfusion bioreactor	Ewing sarcoma TC-71	Shear stress dependency of IGF1 production and Ewing sarcoma sensitivity to IGF-1R inhibitors	^69^
		Up to 8 mPa	Perfusion bioreactor	Ewing sarcoma TC-71	Shear stress-dependent IGF1 secretion by Ewing sarcoma cells	^70^

^a^ The values are listed for the pathological state of bone tissue (i.e., osteosarcoma).

^b^ Human cell lines are listed unless stated otherwise.

PLMA, methacryloyl platelet lysates; bMSC, bone marrow-derived mesenchymal stem cell; HAp, hydroxyapatite; PEGDA, polyethylene glycol diacrylate.

Comparing numerical values of biomechanical stimuli, it seems that there is a significant discrepancy between *in vivo* and 3D *in vitro* values: bone tumors are in general stiffer than utilized scaffolds in models, whereas compression applied *in vitro* is greater. In contrast, values of shear stresses vary across the models but appear to be set around the value of shear stress found in tumors *in vivo*.

As demonstrated, studies on bone tumor engineering have not yet bridged the “*in vivo–in vitro*” gap—from the biomechanics point of view, at least. The reasons may lay in the lack of knowledge about the real physiological and pathophysiological parameters of bone tumor microenvironment, in the complexity of bone tissue *per se*, and in the still emerging understanding of mechanobiological cues.

However, there is clear evidence that the research in this field will be evolving fast in the nearest future. This is mainly driven by the fact that all the observed biomechanical stimuli affect the tumor aggressiveness (invasiveness and ability to metastasize) and chemosensitivity. In turn, relevant drug response is one of the main aims of tumor engineering studies, and therefore, the presence of biomechanical stimuli is urgently required.

For the future perspective, there is still a question to address: what are the most appropriate biomechanical stimuli to be included in osteosarcoma models and how should different stimuli be combined in a reproducible and simple manner? This question can only be answered with the continuation of extensive research on this topic and by overcoming the scarcity of knowledge about the osteosarcoma tumor itself.

## Authors' Contributions

I.B. and K.M.: writing—original draft, visualization. B.O. and L.R.: conceptualization, writing—review and editing, funding acquisition. All authors reviewed the article and agreed to its published version.
